# Glycan remodelling of live adult *Schistosoma mansoni* worms by chemical inhibition of α-mannosidases

**DOI:** 10.1016/j.ijpara.2025.11.002

**Published:** 2025-11-19

**Authors:** Eleonore B. Kuhlemaijer, Tom Veldhuizen, Isabella A. van Hulst, Tessa Liesker, Benjamin J. Hulme, Josephine E. Forde-Thomas, Gabriel Rinaldi, Karl F. Hoffmann, Angela van Diepen, Cornelis H. Hokke

**Affiliations:** a Leiden University Center for Infectious Diseases, Leiden University Medical Center, P.O. Box 9600, Leiden 2300 RC, Netherlands; b Department of Life Sciences (DLS), Aberystwyth University, Aberystwyth, Wales, SY23 3DA, UK; c Department of Biology, University of Oxford, Oxford, OX1 3SZ, UK

**Keywords:** *Schistosoma mansoni*, N-glycans, α-mannosidase inhibition, adult worms

## Abstract

Schistosomiasis is a neglected tropical disease affecting over 250 million people worldwide. *Schistosoma* parasites can survive in the human host for years due to their capability to evade and modulate host immune responses. Glycans and glycoproteins produced by schistosomes are thought to play an important role in shaping parasite-host interactions. In addition, N-linked glycans are vital post-translational protein modifications involved in fundamental cellular and developmental processes like protein folding and cell-cell interactions. In this study, we generated live *Schistosoma mansoni* adult worms with altered N-glycosylation using the α-mannosidase inhibitors kifunensine and swainsonine, compounds which restrict complex N-glycan processing thereby preventing complex N-glycan formation. We show that *ex vivo* cultured adult schistosomes display a stable N-glycosylation profile during two weeks of culture characterised by roughly one-third mannose and two-third complex glycans. Inhibition of α-mannosidases during culture resulted in a modified N-glycan profile over time: kifunensine-treated parasites were found to contain 76% mannose N-glycans, while swainsonine-treated parasites showed a strongly increased abundance of hybrid glycans and (fucosylated) mannose glycans. These observations are in line with the expected effect of the inhibitors on endoplasmic reticulum and Golgi α-mannosidases, respectively. Additionally, we examined the N-glycan composition of the worm tegument, a major parasitic structure found at the parasite-host interface. The tegument contained mainly complex N-glycans, often carrying the GalNAcβ1-4GlcNAc (LacdiNAc, LDN) motif, different from the rest of the schistosome body. Again, α-mannosidase inhibition changed the N-glycosylation profile of the tegument, similarly as observed for whole worms. No negative effects of any of the treatments on parasite motility or morphology were observed, indicating that the basic biology of the worms in culture was not affected by these N-glycosylation changes. Our results demonstrate the feasibility of creating a live glyco-remodelled parasite, setting the scene for studying functional parasite glycobiology and glycan-mediated effects in host-parasite interaction.

## Graphical abstract

**Graphical Abstract:**
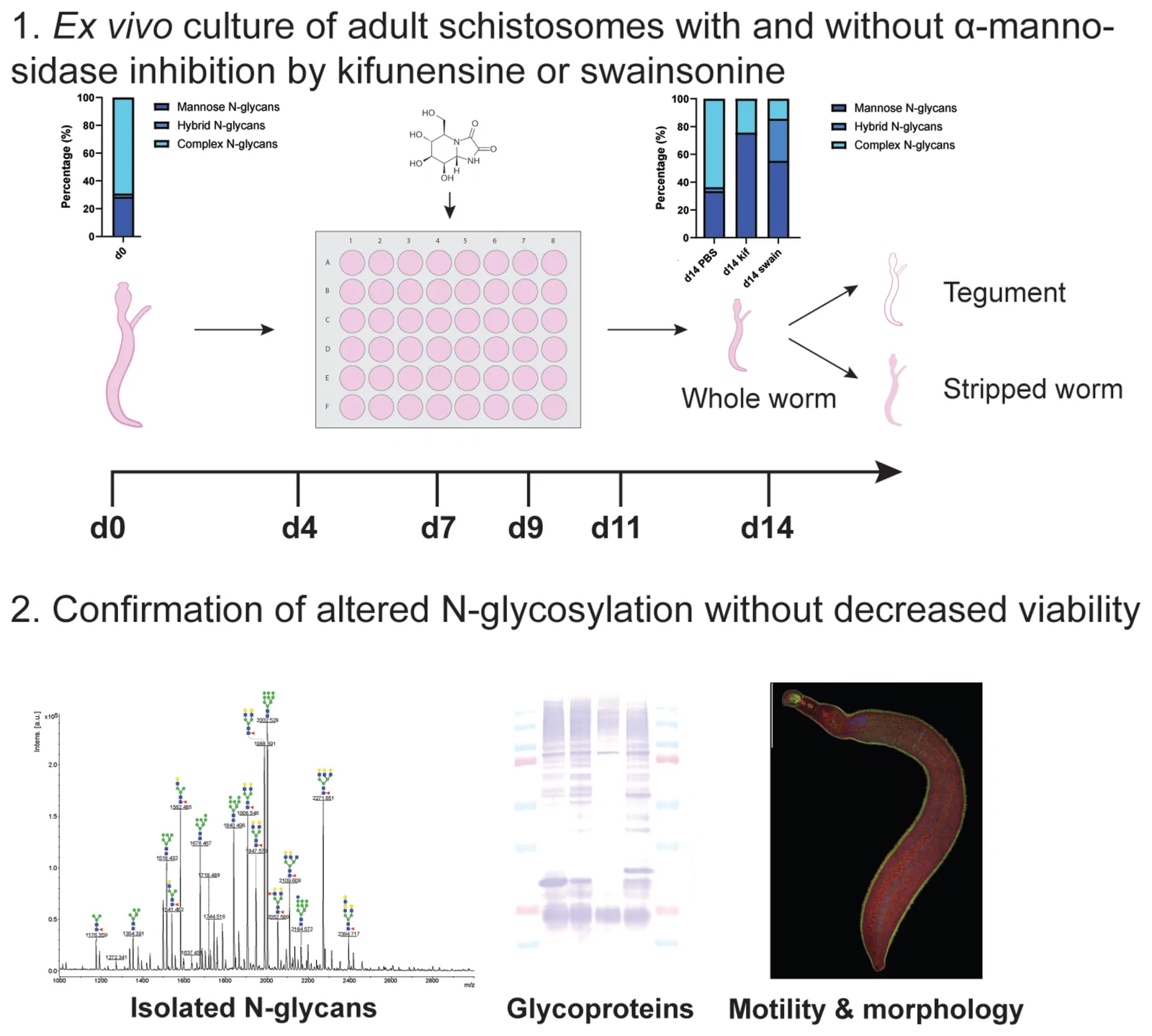

## 1 Introduction

*Schistosoma mansoni* is a parasitic flatworm that causes schistosomiasis in at least 250 million people (Hotez et al., 2014), with an estimated 779 million people at risk of infection (Steinmann et al., 2006). Schistosomiasis mostly affects people living in poor hygienic circumstances with lack of sanitation, leading to chronic infections and poverty, and it classifies as a major neglected tropical disease (King and Dangerfield-Cha, 2008). Although the only available drug praziquantel (PZQ) kills adult worms and is being successfully used in mass drug administration programs, PZQ does not protect against reinfection (Xiao et al., 1985; Sabah et al., 1986; Aboagye and Addison, 2023). Besides, prophylactic vaccines against the disease are lacking and the development of naturally acquired immunity is rare. A thorough understanding of mechanisms underlying parasite survival inside the host is essential in the pursuit of prophylactic vaccines and/or drugs that can protect against (re)infection.

Schistosomes are multicellular eukaryotic organisms, which makes understanding of parasite development and the parasite-host interaction challenging. Carbohydrate chains linked to proteins, known as N- or O-glycans, or to lipids, known as glycosphingolipids (GSLs) (Varki and Kornfeld, 2017), are essential in many eukaryotic biological processes. These glycans have structural functions and are involved in biological processes such as protein folding, cell-cell and cell-matrix interactions and discrimination between self and non-self (Gagneux et al., 2017). Aberrant glycosylation can cause developmental disorders, as is known in human patients (Stepien et al., 2020; Hennermann et al., 2022; Francisco et al., 2023), but has also been shown in experimentally tractable model organisms such as *C. elegans* (Struwe et al., 2009; Katoh et al., 2013; Stevens and Spang, 2013). Glycosylation may, thus, be relevant in parasite development and survival.

Glycans are also well-known pathogen-associated molecular patterns (PAMPs) that can be recognised by pathogen recognition receptors (PRRs) as part of the innate immune system of the host. Interestingly, schistosomes express several distinct glycan motifs, with each developmental stage being characterised by a different glycosylation profile (Smit et al., 2015). For example, N-glycans of *S. mansoni* cercariae and schistosomula are distinguished by Lewis X (LeX; Galβ1-4(Fucα1-3)GalNAc) motifs and core β1-2xyloses, while adult worm glycosylation is marked by a high proportion of LacdiNAc (LDN; GalNAcβ1-4GlcNAc) structures. Eggs, produced by adult female worms, generate N-glycans with a core α1-3fucose and β1-2xylose (Smit et al., 2015). Additionally, schistosome GSL glycans are often highly fucosylated. Cercarial GSL glycans contain multiple single fucoses attached to the glycan backbone and the schistosome-specific Fucα1-2Fuc element is highly abundant in egg GSL glycans and, to a lesser extent, in worms (Smit et al., 2015). Overlap between the schistosome life-stage-specific and host-specific glycan motifs suggests that schistosomes use molecular mimicry to prevent immune attack and interfere with, or hijack, endogenous processes (Schmidt, 1995; Wuhrer et al., 2000; van Diepen et al., 2012). Furthermore, schistosome glycans are ligands for C-type lectin receptors (CLRs) which may lead to host immune skewing (Faveeuw et al., 2003; van Die et al., 2003; van Liempt et al., 2007; Everts et al., 2012; Meevissen et al., 2012; Kuipers et al., 2020). Many schistosome glycans are also targets of host antibodies, which have been suggested to be protective, but may also act as a smoke screen (Eberl et al., 2001; Nyame et al., 2003; Kariuki et al., 2008; Yang et al., 2019). Hence, interplay between schistosome glycans and the host may be fundamental for parasite survival during infection.

Currently, there are limited approaches available for studying the contribution of parasite glycans in parasite development and parasite-host interactions. Therefore, this study aims to generate live glyco-remodelled schistosomes through chemical manipulation of the parasitic N-glycosylation pathway. N-glycosylation is a tightly regulated enzyme-based process in which trimming and subsequent extension of a conserved precursor structure Glc3Man9GlcNAc2 leads to diverse glycan types sharing a common Man3GlcNAc2 core (Stanley et al., 2017). Basic N-glycosylation pathways are largely conserved between eukaryotic organisms, and the enzymes involved and possible routes to manipulate the pathway outcome are well known. This allows for glycan remodelling of cells and possibly of multicellular organisms. In this study, we employed the chemical inhibitors kifunensine and swainsonine, which inhibit the N-glycosylation enzymes α-mannosidase I and α-mannosidase II, respectively. Kifunensine prevents endoplasmic reticulum (ER) and Golgi α-mannosidase I from trimming down Man9GlcNAc2, leading to an accumulation of high mannose glycans in various systems (Elbein et al., 1990; Hering et al., 2005). Swainsonine acts on Golgi mannosidase II, which typically removes mannoses from the intermediate structure GlcNAcMan5GlcNAc2, an essential step for the generation of complex N-glycans, and additionally affects lysosomal and cytosolic mannosidases (Tulsiani et al., 1982; Dorling et al., 1983; Tulsiani and Touster, 1987). By applying these inhibitors in *ex vivo* cultures of adult worms, we aimed to alter the N-glycan profile of *S. mansoni* adult worms. For this, we investigated the schistosome’s isolated glycans as well as its (glyco)proteins and assessed whether these inhibitors caused any effects on parasite motility and morphology. Taken together, our results demonstrate the possibility of generating glyco-remodelled schistosomes that are characterised by an altered N-glycosylation without affecting *ex vivo* parasite viability.

## 2 Materials and methods

### 2.1 Worm culture

*S. mansoni* worms (Puerto Rican-strain) were perfused from infected golden Syrian hamsters (HsdHan:Aura) six to seven weeks post infection, in accordance with the Guide for the Care and Use of Laboratory Animals of the Institute for Laboratory Animal Research and have received approval from the Dutch ‘Central Authority for Scientific Procedures on Animals’ (CCD, License number: AVD11600202215904). The parasites were washed three times with an excess volume of Dulbecco’s Modified Eagle Medium (DMEM; high glucose, no glutamine; Gibco, Paisley, UK) supplemented with antibiotic antimycotic solution (ABAM; 2x concentrated, Sigma-Aldrich, Saint Louis, MO, USA). Blood clots and hamster hairs were removed before the worms were incubated for 45 minutes at 37 °C. After incubation, the worms were divided over culture plates or flasks with a disinfected paint brush. During this transfer, the parasites were inspected under a dissection microscope to ensure an equal ratio of female and male worms and to prevent the inclusion of damaged or dead worms. Worms were cultured aseptically in serum-free culture medium, consisting of hybridoma-SFM (Gibco) supplemented with ABAM (2x concentrated), ascorbic acid (200 µM, J.T. Baker, Phillipsburg, NJ, United States), human red blood cells (0.005 – 0.01% (v/v), Sanquin, the Netherlands) and chemically defined lipid concentrate (Gibco). One worm pair, consisting of one female and one male, was cultured in one well of a tissue culture-treated 48-well culture plate (Corning) in 1 mL culture medium, or thirty worm pairs were cultured in a tissue culture-treated T75 culture flask with vent cap (Corning) in 10 – 15 mL culture medium. Medium was refreshed every two to three days and new compounds were added. During culture, the worms were continuously treated with either phosphate-buffered saline (PBS; Frensius Kabi, Bad Homburg, Germany) as PBS-control, kifunensine (21.5 µM; Cayman Chemical, Ann Arbor, MI, USA) or swainsonine (25 µM; Biotechne, Minneapolis, MN, USA or Cayman Chemical). After a maximum of 14 culture days, the worms were harvested, washed with PBS three times and stored at -20 °C. Worms used for N-glycan analysis were stored in PBS, while worms destined for protein analysis by SDS-PAGE and western blotting were stored dry.

### 2.2 Tegument isolation

The isolation of the adult worm tegument was performed as described previously (Timson, 2020). Briefly, the harvested and washed worms were snap-frozen in liquid N_2_ before storage at -20 °C. After thawing, the worms were washed in 10 mM Tris–HCl (pH = 7.4, Thermo Fisher Scientific, Waltham, MA, USA) supplemented with 0.84% (w/v) NaCl (Merck, Darmstadt, Germany). Next, the worms were incubated for five minutes in 10 mM Tris–HCl (pH = 7.4). After five short vortex bursts, the supernatant was separated from the remaining worms (which were retained for further processing) and centrifuged at 17,000 × *g*. After this, the supernatant was discarded, leaving the tegumental pellet for further processing.

### 2.3 N-glycan analysis

Four to six worms were potter-homogenised, sonicated for 20 minutes in an ultrasonic bath (45 kHz; VWR, Radnor, PA, USA), lyophilised, resuspended in PBS and sonicated again. Next, the mixture was brought to 1.3% (w/v) sodium dodecyl sulfate (SDS; Merck) and 0.1% (v/v) β-mercaptoethanol (VWR) and incubated for ten minutes at 95 °C to denature proteins. After cooling down to room temperature (RT), the mixture was neutralised with 1.3% (v/v) Nonidet P40 substitute (Sigma-Aldrich) and incubated with 0.02 U/µL peptide-N-glycosidase F (PNGase F; Roche, Basel, Switzerland) for 24 hours at 37 °C while shaking. The released N-glycans were isolated through column purification, labelled and purified by bio-gel, as described previously (Smit et al., 2015). In short, C18 cartridges (J.T. Baker) were conditioned with acetonitrile (ACN; Biosolve, Valkenswaard, the Netherlands), followed by 60% (v/v) ACN and 0.1% (v/v) trifluoroacetic acid (TFA; Merck) and milliQ (MQ) water. After sample application, the column was washed with 10% ACN and MQ water, while the flow-through was collected. This flow-through was then used for carbon SPE purification. The carbon SPE cartridges (Merck) were conditioned with ACN, 50% ACN and 0.1% TFA and MQ water. Subsequently, the sample was applied to the column and washed with MQ water. Glycans were then eluted with 25% ACN followed by 50% ACN and 0.1% TFA and concentrated in a vacuum concentrator. After the isolated glycans were redissolved in MQ water, the glycans were labelled with 48 mg/mL anthranilic acid (AA; Sigma) in a mixture (3:10) of glacial acetic acid (Merck) in dimethyl sulfoxide (DMSO; Sigma-Aldrich) and 107 mg/mL 2-picoline borane complex (Sigma-Aldrich). For bio-gel purification, 100 mg/mL bio-gel P-10 (Bio-Rad, Hercules, CA, USA) in 10% ACN was added to 0.45 µm wwPTFE filter plates (Pall Life Sciences, Port Washington, NY, USA). The bio-gel was conditioned with MQ water and 80% ACN. Labelled glycans were brought to a content of 80% ACN and applied to the gel. After washing with 80% ACN, the labelled glycans were eluted with MQ water and concentrated. Lastly, the labelled glycans were spotted on a 384-well polished steel target plate (Bruker, Billarica, MA, USA) for matrix-assisted laser desorption/ionisation (MALDI). To this end, 1 µL of 20 mg/mL 2,5-dihydroxybenzoic acid (DHB; Bruker) in 30% ACN was applied to the plate. Then, 1-2 µL of the labelled glycans was spotted, followed by another droplet of 1 µL of DHB in 30% ACN. Additionally, enzymatic digestions with exoglycosidases were performed on the labelled glycans. For this, β-N-Acetylhexosaminidasef (cat. no. P0721S, New England Biolabs (NEB), Ipswich, MA, USA) and β1-4 Galactosidase S (cat. no. P0745S NEB) were used according to the manufacturer’s instructions with minor adjustments: 1 µL enzyme was added and the digestion was incubated overnight at 37 °C. The hexosaminidase was used in combination with hydrofluoric acid (HF; Riedel-de-Haën, Seelze, Germany) treatment prior to enzymatic digestion, as described before (Meyer et al., 2007). The HF-treated glycans were redissolved in MQ water. The digested labelled glycans were spotted on the target plate by C18 ziptip. To that end, the C18 ziptip (ZipTip 0.2 µL C18 resin, Merck) was washed twice with 50% ACN/0.1% TFA in MQ water, followed by two washes with 0.1% TFA in MQ water. Then, the sample was pipetted five to ten times over the ziptip and the ziptip was washed three times with 0.1% TFA in MQ water. Lastly, the sample was eluted with 1 – 2 µL 10 mg/mL DHB in 50% ACN/0.1%TFA in MQ water on the target plate.

### 2.4 MALDI-TOF MS

The isolated, purified, AA-labelled N-glycans were analysed by matrix-assisted laser desorption/ionisation time-of-flight (MALDI-TOF) mass spectrometry (MS) on a rapifleX mass spectrometer (Bruker) with flexControl software (version 4.0; Bruker). To start, the instrument was calibrated using the peptide calibration standard II (Bruker). Next, samples were measured in negative mode between 1000 and 3000 m/z. The obtained mass spectra were analysed with flexAnalysis software (version 4.0; Bruker) and peaks with a minimum signal-to-noise (S/N) ratio of three were manually annotated, as described before (Petralia et al., 2022). Based on the mass-to-charge (m/z) ratio of the annotated peaks, possible glycan structures were acquired using the GlycoPeakfinder tool of GlycoWorkBench (Ceroni et al., 2008), as described previously (Petralia et al., 2022). The annotation of N-glycan compositions was based on published mass spectra of *S. mansoni* adult worms (Smit et al., 2015) and on exoglycosidase digestions. Finally, the S/N ratio of identified glycans was normalised to the total S/N ratio of all identified glycans per sample, giving the relative presence of each glycan as a percentage.

### 2.5 Graphical representation and statistical analysis of MALDI-TOF MS data

Graphs of the glycan percentages were made in GraphPad (Boston, MA, USA). The bar graphs visualising the distribution of complex, hybrid and mannose N-glycan classes were created based on the medians of the two most abundant classes, after which the least abundant class was calculated as 100% minus the two medians. Non-parametric statistical tests to evaluate whether the relative presence of glycan structures differed significantly between treated and PBS-control parasites were performed in GraphPad. For each timepoint, a comparison between kifunensine and PBS and/or swainsonine and PBS was made. When two groups were compared, a Mann Whitney U test was performed and the exact significance was reported. In case of three groups, a Kruskal-Wallis test was used, followed by a post-hoc test with Dunn’s correction in which kifunensine and swainsonine were compared to the PBS control group.

### 2.6 SDS-PAGE and western blot

Whole worms, tegument and stripped worms were dissolved in Laemmli sample buffer with 6% (w/v) DTT, vortexed and incubated at 95 °C for ten minutes. This procedure was repeated once or twice, until the worms or tegument were fully dissolved. Next, an equivalent volume of one or two worms (whole worms), 20 worms (tegument) or two worms (stripped worms) was loaded alongside a Precision Plus Protein Dual Color Standards (Bio-Rad, Hercules, CA, USA) on a 10% polyacrylamide gel and separated at 100 V for ~90 minutes by sodium dodecyl sulfate–polyacrylamide gel electrophoresis (SDS-PAGE). Then, the proteins were transferred to a methanol-activated (Supelco, Merck) 0.45 µm pore size polyvinylidene fluoride (PVDF) membrane (GE Healthcare, Chicago, IL, USA) at 100 V constant voltage for 90 minutes. Additionally, an SDS-PAGE gel was stained with colloidal blue (Invitrogen) according to the manufacturer’s instructions to visualise the separated proteins. To prevent non-specific binding of antibodies on the western blot, the membrane was blocked with tris-buffered saline (TBS) supplemented with 0.1% (v/v) Tween-20 and 0.5% (w/v) bovine serum albumin (TBSTB) overnight at 4°C. After blocking, the membrane was incubated with the primary antibody diluted in TBSTB for one hour at RT. Monoclonal antibodies were in-house produced as described before (van Dam et al., 1993; Deelder et al., 1996; Nibbeling et al., 1998). The antibodies 100-4G11 (trimannosyl), 273-3F2-A (LDN),128-1E7-C and 291-5D5-A (fucosylated LDN) were used. Primary antibody incubation was followed by three wash steps in TBST and incubation with an anti-mouse polyvalent immunoglobulins (G,A,M)−alkaline phosphatase antibody (1:1000; produced in goat; Sigma-Aldrich) in TBSTB for one hour at RT. Lastly, the membrane was washed four times in TBST, once in TBS and signal was visualised through incubation with 5-Bromo-4-chloro-3-indolyl-phosphate (BCIP; Roche) and 4-nitro blue tetrazolium chloride (NBT; Sigma-Aldrich), dried and imaged with a Scanjet G3110 scanner (HP, Palo Alto, CA, USA) with Windows Picture Acquisition software.

### 2.7 Motility scoring

Worm motility was assessed through scoring as described previously (Ramirez et al., 2007). In short, during the first four culture days and before every medium replacement, worm motility was scored on a scale from four to zero (4 = normally active; 3 = slowed activity; 2 = minimal activity, occasional movement of head and tail; 1 = absence of motility apart from gut movements; 0 = total absence of mobility) (Ramirez et al., 2007).

### 2.8 Staining with live dyes and confocal microscopy

Worms were cultured in the same culture conditions as described above, although in medium consisting of DMEM supplemented with 10% (v/v) heat-inactivated foetal bovine serum (hiFBS; Serana, Brandenburg, Germany), 1M HEPES (Sigma-Aldrich) and 200 mM L-glutamine (Sigma-Aldrich). At the last day of culture, CellMask Green Actin Tracking Stain (1:3000; Invitrogen, Waltham, MA, USA), CellMask Plasma Membrane Stain Deep Red (1:3000; Invitrogen) and NucBlue Live ReadyProbes Reagent (two droplets; Invitrogen) were added followed by overnight incubation. The next day, the worms were washed with PBS, fixed with 4% (v/v) formaldehyde (Sigma-Aldrich) overnight and washed with PBS. Subsequently, the worms were mounted with Fluoromount-G™ Mounting Medium, with DAPI (Invitrogen) on adhesive glass microscopy slides (SuperFrost, Knittel Glass, Braunschweig, Germany) and covered with a glass cover slip (Menzel-Gläser, Braunschweig, Germany). Mounted worms were then imaged with a Leica true confocal scanning (TCS) SP8 white light laser (WLL) microscope (Leica Microsystems, Wetzlar, Germany) with Leica Application Suite (LAS) X software (version 3.5.7.23225; Leica Microsystems). NucBlue (excitation: 405 nm, emission: 410 - 490 nm), CellMask Green (excitation: 495 nm, emission: 500 - 648 nm) and CellMask Deep Red (excitation: 653 nm, emission: 658 - 777 nm) were visualised through line sequential scanning with a 63x objective (Leica HC PL APO CS2 63×/1.40na OIL). Individual images of each worm were merged into one image through the LAS X Navigator application and a 500 µm scale bar added to each image.

## 3 Results

### 3.1 Shifting N-glycosylation of adult schistosomes by *ex vivo* culture with and without α-mannosidase inhibition

**Figure 1:**
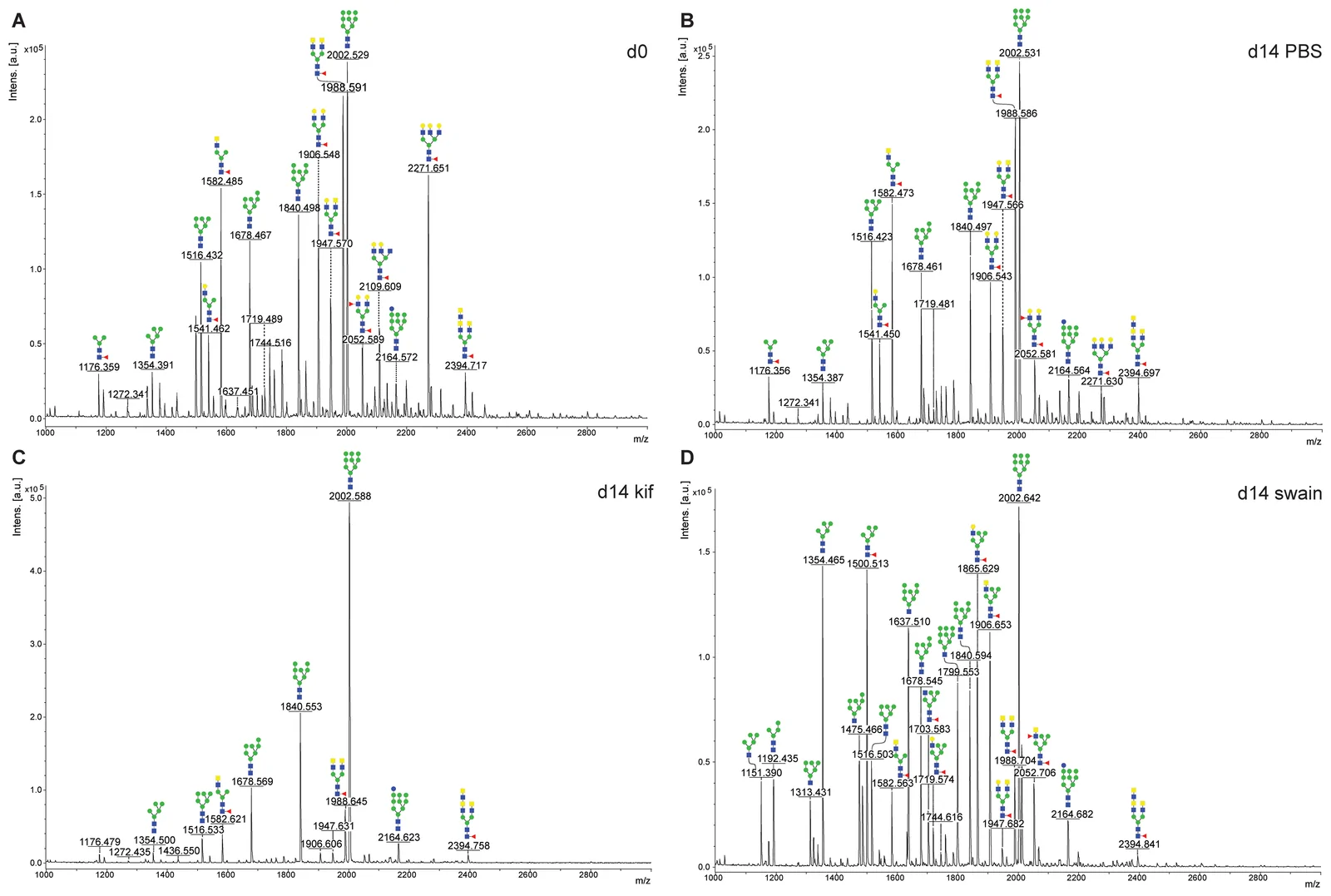
MALDI-TOF MS profiles of N-glycans from adult schistosomes directly after perfusion (d0) and after *ex vivo* culture (d14) with or without chemical inhibition of α-mannosidases AA-labelled PNGase F-released N-glycans measured by MALDI-TOF MS from worm pairs at d0 (A) and at d14 with PBS-control (B), kifunensine- (C) or swainsonine-treatment (D). Blue square: N-acetylglucosamine; green circle: mannose; yellow square: N-acetylgalactosamine; yellow circle: galactose; red triangle: fucose. For each condition a representative mass spectrum of one of four biological replicates is shown.

As changes in N-glycosylation might occur during *ex vivo* parasite culture, we first studied the N-glycan composition of adult schistosomes after 14 days of *ex vivo* culture and compared this to freshly collected parasites. PNGase F-sensitive glycans were released from worm pairs harvested at day (d0) and d14 and analysed by MALDI-TOF MS. The obtained mass spectra showed the presence of various glycan types, with Man9GlcNAc2 (M9; Supplementary figure 1) being the most abundant structure at both d0 and d14 (Figure 1 A & B). Other prevalent mannose glycans included Man8GlcNAc2 (M8), Man7GlcNAc2 (M7) and Man6GlcNAc2 (M6) (Supplementary figure 1). In addition, several complex N-glycans were found abundantly expressed, including structures with the antenna motifs LDN and LacNAc (LN; Galβ1–4GlcNAc). However, two compositions that were highly present in the spectrum of d0 pairs (H5N5F1 and H6N5F1, Figure 1 A) were found in a lower relative amount in worm pairs at d14 (Figure 1 B). These N-glycans contained two or three LN antenna, respectively. Multi-antennary LN-containing N-glycans are known to be present on *S. mansoni* egg proteins (Khoo et al., 1997), which are still present in the female body directly after perfusion, and have been reported as female-specific before (Wuhrer et al., 2006). Therefore, we assessed the adult worm sex-specific N-glycan structures before and after culture by isolating the N-glycans from females and males separately at d0 and 14. These worms were cultured as pairs but separated during harvesting. Indeed, we found the structures H5N5F1 and H6N5F1 almost exclusively in female samples at d0 (Supplementary figure 2 A). Furthermore, the N-glycan structures H3N4F1 and H3N6F1, containing one or two LDN motifs respectively, were found to be male-specific at d0 (Supplementary figure 2 B). Yet, the sex-specific differences were reduced at d14; in female samples H5N5F1and H6N5F1 were reduced and H3N4F1 and H3N6F1 were increased, while in male samples, H3N4F1 and H3N6F1 were decreased (Supplementary figure 2 A & B). The distribution of mannose, hybrid and complex glycans was comparable between female and male, both at d0 and d14 (Supplementary figure 2 C). Together, these data indicate that *S. mansoni* adult worm N-glycosylation is largely stable during *ex vivo* culture, with some sex-specific differences. Next, we analysed the effect of α-mannosidase inhibition by kifunensine and swainsonine on the N-glycans of adult schistosomes (Figure 1 C & D). At d14, N-glycans of kifunensine-treated worms contained increased levels of mannose glycans, of which M9 was the most common structure, followed by M8 and M7 (Figure 1 C & Supplementary figure 3). Swainsonine treatment, on the other hand, led to hybrid structures with a complex motif on one branch and a trimannosyl element on the other (Figure 1 D & Supplementary figure 3), as confirmed by exoglycosidase digestion (Supplementary figure 4). M9 was again the structure with the highest intensity in the N-glycan profile, followed by Man5GlcNAc2 (M5) and fucosylated M5 (Figure 1 D). In addition to these N-glycans with a Man3GlcNAc2 core structure, we also found mannosylated glycans with only one core GlcNAc in swainsonine-treated worms (Figure 1D). These glycans are not attached to proteins, and are therefore known as free N-glycans (FNGs). Again, no clear differences between the N-glycan composition of females and males were found (Supplementary figure 2 A & D). Thus, chemical inhibition of α-mannosidases during *ex vivo* culture of adult worms generated a clear shift towards M7-9 glycans in kifunensine-treated and hybrid and (fucosylated) M5 glycans in swainsonine-treated worms.

### 3.2 Dynamics of altered N-glycosylation in adult schistosomes over time

**Figure 2:**
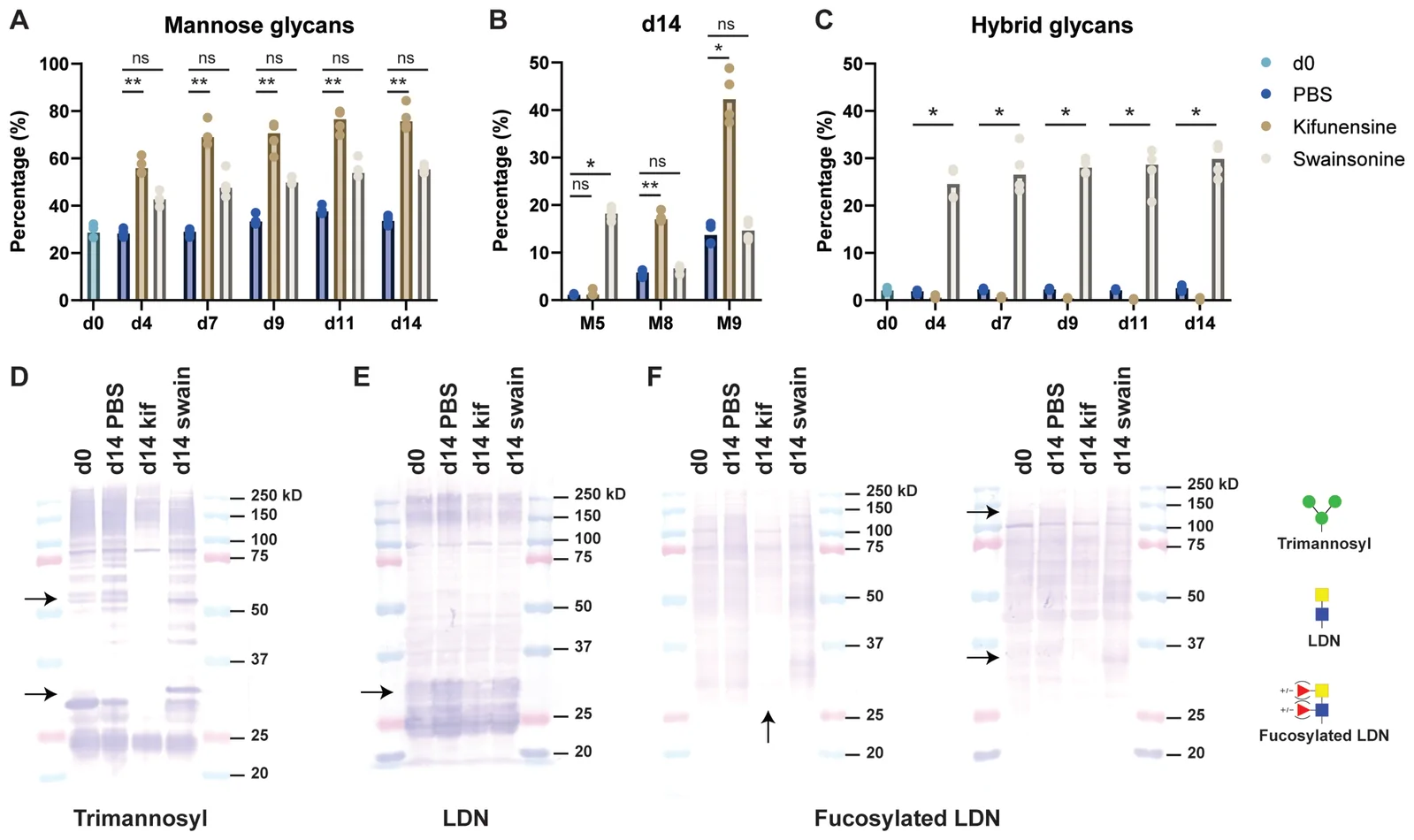
Composition of N-glycans in freshly collected, PBS-control and α-mannosidase inhibitor-treated adult schistosomes AA-labelled PNGase F-released N-glycans were measured by MALDI-TOF MS and the relative abundance of mannose (A), Man5GlcNAc2 (M5), Man8GlcNAc2 (M8) and Man9GlcNAc2 (M9) (B) and hybrid (C) structures was calculated for freshly collected worms (d0) and at the indicated time points for each treatment (d4, d7, d9, d11, d14). * p < 0.05 ** p < 0.01. Data of four biological replicates is shown. Additionally, antibodies against the glycan motifs trimannosyl (Manα1-3(Manα1-6)Man), LDN (GalNAcβ1-4GlcNAc) or fucosylated LDN (GalNAcβ1–4(Fucα1–3)GlcNAc; LDN-F, Fucα1–3GalNAcβ1–4GlcNAc; F-LDN and Fucα1–3GalNAcβ1–4(Fucα1–3)GlcNAc; F-LDN-F) were used to detect these motifs in glycosylated proteins (D-F). Arrows indicate differences in protein bands between different samples. Representative blots of two biological replicates are shown.

Next, we examined in more detail the dynamics of N-glycosylation changes induced by kifunensine and swainsonine during the two-week culture period. Accordingly, samples were taken before treatment (d0) and at d4, d7, d9 and d14 (Supplementary figure 1 A) and PNGase F-released N-glycans were analysed by MALDI-TOF MS. Detected N-glycans were categorised as complex, mannose or hybrid glycans (Supplementary figure 1 B). At d0, the median percentage of mannose N-glycans compared to all N-glycans was 28.6% (Figure 2 A). Over time, this percentage stayed constant in PBS-control worms but increased in kifunensine- as well as swainsonine-treated worms. At d4, kifunensine-treated worms had 55.8% mannose glycans and swainsonine-treated worms contained 42.6%, both significantly more than the 28.2% mannose glycans in PBS-control worms. The mannose glycans expanded further by kifunensine as well as swainsonine treatment, up to 75.6% in kifunensine-treated and 55.3 % in swainsonine-treated schistosomes at d14, compared to 33.5% mannose glycans in PBS-control (Figure 2 A). While M8 and M9 where the main mannose glycans in kifunensine-treated worms with 17.0% and 42.2% respectively, (fucosylated) M5 appeared to be specific for swainsonine-treatment with a median 18.2% (Figure 2 B). Moreover, swainsonine-treatment resulted in a very strong rise of hybrid N-glycans, from 2.3% at d0 up to 30.2% at d14, compared to 2.5% in PBS-control worms and 0.3% in kifunensine-treated worms at d14 (Figure 2 C). To achieve more insights into the effects of kifunensine- and swainsonine-treatment at glycoprotein level, we performed western blots with antibodies specific for the motifs trimannosyl (Manα1-3(Manα1-6)Man), LDN and fucosylated LDN (GalNAcβ1–4(Fucα1–3)GlcNAc; LDN-F, Fucα1–3GalNAcβ1–4GlcNAc; F-LDN and Fucα1–3GalNAcβ1–4(Fucα1–3)GlcNAc; F-LDN-F) (Figure 2 D - F & Supplementary figure 4). Proteins from d0 and d14 PBS-control showed comparable banding patterns for all antibodies. The trimannosyl motif, present in M3 and M5, was less abundantly detected in kifunensine-treated worms than in PBS-control worms, specifically in proteins between 25 and 75 kD (Figure 2 D). However, swainsonine-treatment resulted in the presence of trimannosylated glycoproteins not detectable in untreated worms. The western blot pattern of LDN was, in general, similar between the different treatments, except for a lower reactivity in kifunensine-treated worms just above 25 kD Mw (Figure 2 E). Lastly, fucosylated LDN was less observed in kifunensine-treated worms when compared to PBS-control and swainsonine-treated samples (Figure 2 F). Collectively, these data demonstrate that α-mannosidase inhibition in adult schistosomes leads to changes in N-glycans, which are amplified over time.

### 3.3 Glyco-remodelled worms do not show apparent changes in motility or morphology

**Figure 3:**
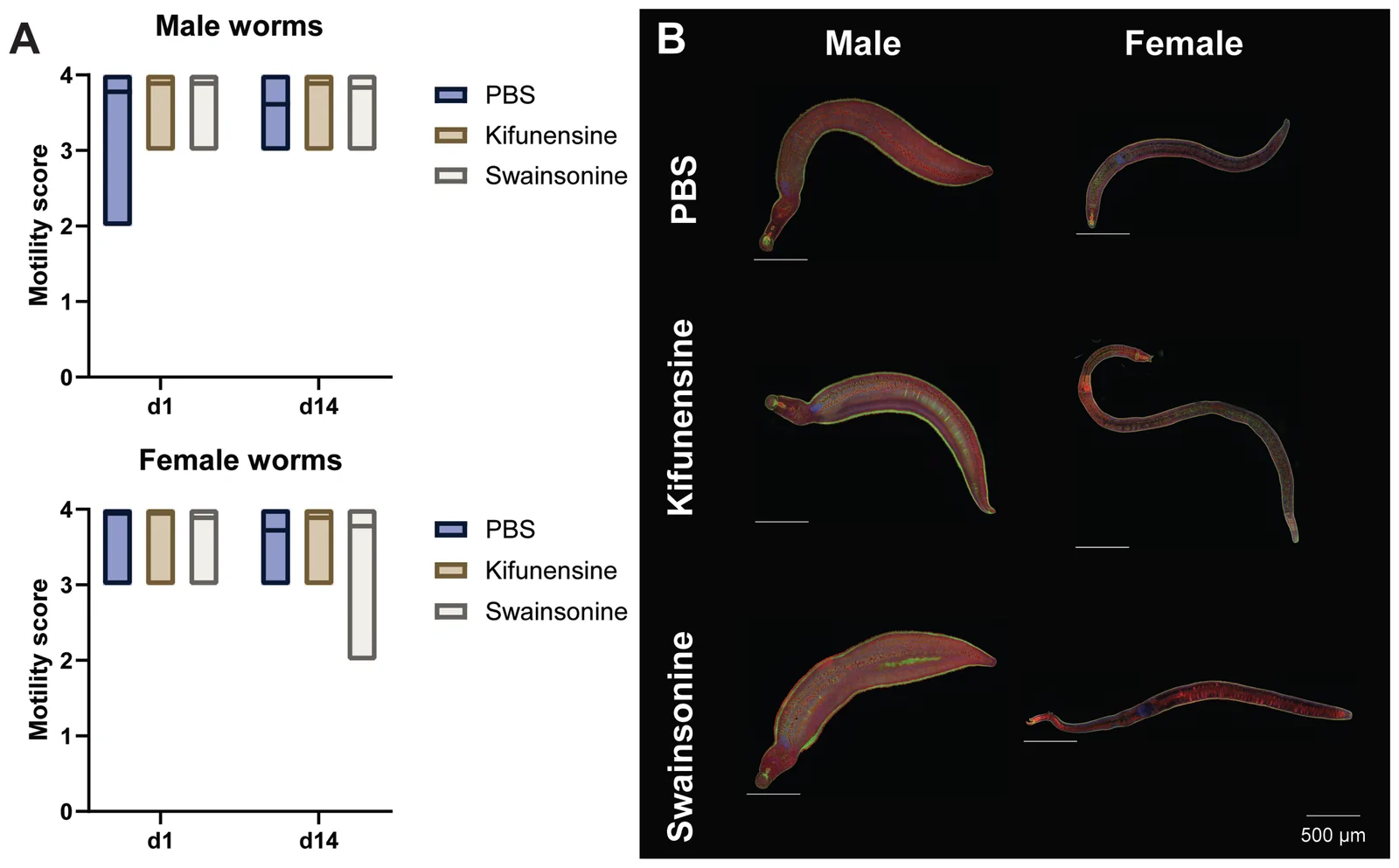
Motility scores and confocal microscopy of PBS-control and α-mannosidase inhibitor-treated adult schistosomes Parasite motility was scored according to the WHO-TDR motility index: 4 = normally active; 3 = slowed activity; 2 = minimal activity, occasional movement of head and tail; 1 = absence of motility apart from gut movements; 0 = total absence of mobility (Ramirez et al., 2007). The score of 18 males (top) and 18 females (bottom) on d1 and d14 across three individual cultures, including the median(A). For each condition, five or six male and female parasites were live stained at d14, fixed and imaged. One representative image is shown. Cell nuclei (blue), membranes (red) and actin (green) are represented (B). Scale bar: 500 µm.

As treatment with the chemical α-mannosidase inhibitors led to altered N-glycans in the parasites, we next tested whether the treatment affected the worms in their motility and/or morphology. To address worm motility, the individual worms were scored based on the WHO-TDR motility index (Ramirez et al., 2007), with 4 as highest and 0 as lowest score. At day 1 of culture, 100% of the female and ≥ 94% of males had a motility score of 3 or 4, indicating normal movement (Figure 3 A). Only one out of 18 PBS-control males had a score of 2 (minimal activity). At day 14, one out of 18 swainsonine-treated females scored a 2, while all other swainsonine-treated females scored 3 or 4. Additionally, all otherwise-treated females and all males had a score of 3 or 4. Second, we examined worm morphology by confocal microscopy. Live worms were stained for nuclei (blue), cell membranes (red) and actin (green), which allowed for visual inspection of anatomical structures, including the oral and ventral suckers, oesophagus, reproductive organs, muscles and tegumental layer. These hallmarks were visually unaffected in the kifunensine- and swainsonine-treatment worms when compared to the PBS-control (Figure 3 B). Together, worm motility and morphology observations did not indicate a kifunensine- or swainsonine-mediated diminished viability or substantial integrity effect in comparison to PBS-controls.

### 3.4 Tegumental N-glycosylation is marked by complex glycans, which is affected by α-mannosidase inhibition

**Figure 4:**
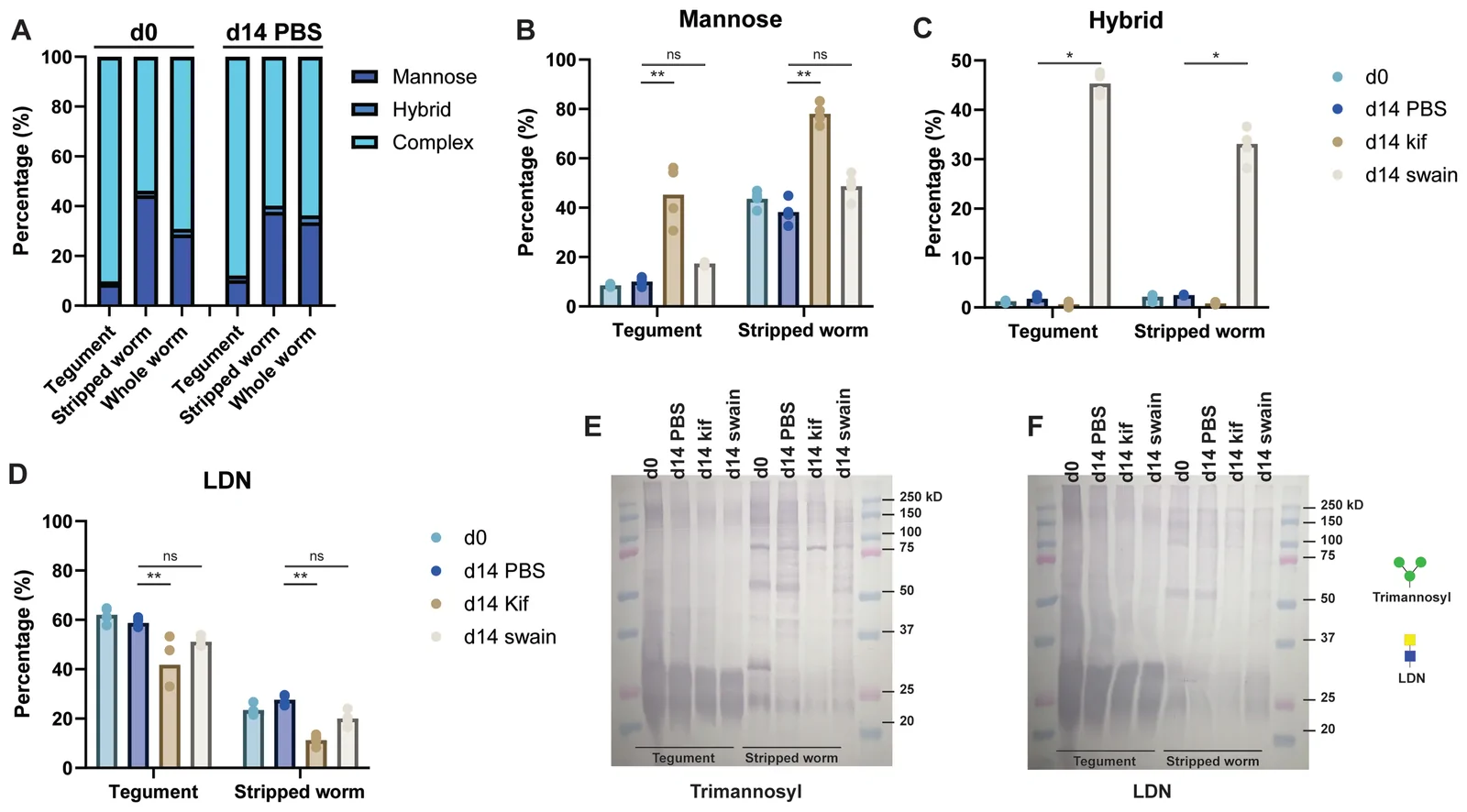
Composition of N-glycans in tegument and stripped worms of freshly collected, PBS-control and inhibitor-treated adult schistosomes AA-labelled PNGase F-released N-glycans were analysed by MALDI-TOF MS to obtain the N-glycan compositions of tegument, stripped worms and whole worms, before and after *ex vivo* culture for 14 days (A). The relative abundance of mannose N-glycans (B), hybrid N-glycans (C) and N-glycans with the LDN motif (D) was depicted for each treatment. * p < 0.05 ** p < 0.01. Data of four biological replicates is shown. In addition, antibodies against the glycan motifs trimannosyl (E) and LDN (F) were used to detect these motifs in glycosylated proteins (D). Representative blots of two biological replicates are shown.

The tegument is the outer layer of the adult schistosome’s body and forms the parasite’s interface with the host. Therefore, we dissected the N-glycan profile of the worm tegument and analysed if N-glycosylation changes in the tegument occur differently or similarly to the whole worm body. For this purpose, we isolated the tegument from mixed-sex pairs and measured the PNGase F-sensitive N-glycans in this tegumental isolate as well as the rest of the worm (‘stripped worm’). First, we found that the tegumental N-glycan profile of freshly collected worms mainly consisted of complex glycans (median of 90.0 %), and only 8.7% mannose glycans (Figure 4 A). In contrast, the profile of stripped worms contained 54.0% complex and 43.5% mannose glycans. After 14 days of culture, the tegumental N-glycans were made up of 87.5% complex glycans and 10.3% mannose glycans (Figure 4 A). In stripped worms, complex and mannose glycans contributed 59.6 and 37.7% to the total N-glycans, respectively. In all fractions, hybrid glycans were between 2 and 3% of all measured N-glycans. Next, we tested the effect of α-mannosidase inhibition on the tegument and stripped worms. Kifunensine led to a significant increase of mannose glycans when compared to PBS-control in both tegument and stripped worms (47.0% and 78.0%, respectively) (Figure 4 B). The hybrid N-glycans in tegument and stripped worms were significantly higher in swainsonine-treated worms compared to PBS-control worms, with 45.8 % in tegument and 33.9% in stripped worms (Figure 4 C). LDN is a relatively abundant motif in adult worm N-glycans. At culture day 14, 62.1% of all tegumental N-glycans and 32.1% of all N-glycans in stripped worms contained at least one LDN motif on their branches (Figure 4 D). Kifunensine treatment lowered these percentages significantly to 41.6% in the tegument and 13.2% in the stripped worms. Western blot analyses using antibodies for trimannosyl and LDN (Figure 4 E & F) were performed to study the N-glycan profiles attached to proteins. The trimannosyl motif was highly abundant in low molecular weight proteins of the tegument, but in stripped worms in high as well as low molecular weight proteins (Figure 4 E). LDN was found mostly in low molecular weight tegumental proteins of all conditions and in smaller amounts in stripped worm proteins of different masses (Figure 4 F). Kifunensine-treated stripped worms displayed a lower abundance of trimannosyl as well as LDN motifs. Taken together, these data show that tegumental N-glycans differ from stripped worm N-glycans, and that kifunensine and swainsonine treatment impacts the tegumental as well as the stripped worm N-glycosylation.

## 4 Discussion

Schistosome N-glycosylation is suggested to be involved in the parasite’s development and survival, but approaches to study N-glycans in living schistosomes are still limited. In this study, we examined the N-glycosylation of *ex vivo* cultured adult schistosomes and employed chemical α-mannosidase inhibition to alter the N-glycans of live parasites. Our results show that the N-glycosylation profiles of inhibitor-treated adult worms are significantly changed, without visually affecting parasite movement or morphology. Additionally, we describe that tegumental N-glycans are characterised by the abundant presence of LDN motifs and confirm that these glycans are affected by α-mannosidase inhibitors as well.

*Ex vivo* glyco-remodelling of schistosomes as performed in this study creates the opportunity to examine N-glycan changes over time. As we have shown in this study, α-mannosidase inhibition led to a swift effect on N-glycosylation with significantly more mannose (kifunensine) and hybrid (swainsonine) type of glycans detectable as soon as four days after initiation of culture. These effects gradually increased over time and were further characterised through use of glycan-specific antibodies, which demonstrated differential binding patterns between protein extracts derived from PBS-control versus inhibitor-treated parasites. In particular, kifunensine-treated parasite extracts contained fewer binding sites for antibodies against trimannosyl, LDN and fucosylated LDN. Kifunensine is a widely used inhibitor of α-mannosidase I (Elbein et al., 1990). Schistosomes treated with this inhibitor showed increased levels of mannose glycans, with M9 and M8 as the dominant types. Swainsonine is known to inhibit Golgi α-mannosidase II, and lysosomal and cytosolic α-mannosidase (Tulsiani et al., 1982; Dorling et al., 1983; Tulsiani and Touster, 1987). After swainsonine-treatment of schistosomes, (fucosylated) M5 as well as hybrid N-glycans were typically seen. As a result of both treatments, complex N-glycans decreased relatively. These results are in concordance with previous reports for *in vitro* cultured cells (Elbein et al., 1990; Zhou et al., 2021; Morikawa et al., 2022). Specifically, transfected LDN- and LDN-F-expressing Lec8 cells used as a model for schistosome cells, did produce lower amounts of these motifs after kifunensine treatment (Prasanphanich et al., 2014). Additionally, kifunensine treatment of *Nicotiana benthamiana* plants, that have also been used for functional characterisation of schistosome glycosyltransferases (van Noort et al., 2020), leads to a similar increase in M9 N-glycans (Roychowdhury et al., 2018).

Interestingly, we also found an increase of FNGs with one instead of two core GlcNAc residues (GlcNAc1 glycans), as well as fucosylated M5, in swainsonine-treated parasites. Both types of glycans have been reported to be specific for swainsonine-treated mammalian cells (Crispin et al., 2006; Yanagida et al., 2006; Chantret and Moore, 2008; Morikawa et al., 2022). The observed GlcNAc 1 FNGs were high-mannose structures (M5-9), which are hypothesised to be present in increased amounts due to inhibition of cytosolic α-mannosidase, in contrast to hybrid and paucimannose (M2-4) N-glycans, resulting from inhibited Golgi α-mannosidase II and lysosomal α-mannosidase, respectively (Morikawa et al., 2022). Further research is needed to understand how swainsonine treatment is associated with increased FNGs in schistosomes more extensively. Together, our findings demonstrate that kifunensine and swainsonine are effective in altering schistosome N-glycosylation and lead to an N-glycan profile which corresponds with findings in other eukaryotes.

Additionally, live parasite glyco-remodelling allows studying the effects of altered glycosylation on parasite development and viability. Aberrant glycosylation and glycosidase defects are often associated with severe disease, such as the lysosomal storage disease alpha-mannosidosis in humans (Hennermann et al., 2022). Kifunensine supresses ER-associated degradation (ERAD) of misfolded proteins and disrupts glycoprotein trafficking (Wang et al., 1997; Tokunaga et al., 2000; Lodge et al., 2020). Moreover, swainsonine induces lysosomal storage disease in cattle, leading to disease affecting neurology, reproduction and immunity (Cook et al., 2009; Cook et al., 2014). Autophagy, apoptosis and paraptosis have been identified as potential underlying mechanisms of swainsonine-poisoning (Wang et al., 2021; Wang et al., 2021). In this study no effects of kifunensine or swainsonine treatment on parasite motility and morphology were observed after two weeks of *ex vivo* culture in the presence of these inhibitors. Confocal microscopy of worms fluorescently stained for nuclei, cell membranes and actin did not demonstrate substantial changes in general morphology (e.g. the ventral and oral sucker, oesophagus and tegument) across treatments. The discrepancy between the reported disease in humans and cattle and our observations could possibly be related to the higher complexity of vertebrates compared to invertebrate organisms. For example, in swainsonine-induced poisoning of cattle, specific cells such as neurons and macrophages are affected, as well as tissues like the kidney, liver and spleen (Chenchen et al., 2014). Although schistosomes possess complex structures such as neurons (Gustafsson, 1987) and schistosome cells contain lysosomes (Nawaratna et al., 2011), a direct comparison between schistosomes and vertebrates in this respect is not possible due to their vastly different biological and molecular make up. Furthermore, maintaining the parasites in laboratory conditions could prevent environmental stressors causing the altered N-glycosylation to become relevant. In *Caenorhabditis elegans*, knock-out of all GnT I (alpha-1,3-mannosylglycoprotein 2-beta-N-acetylglucosaminyltransferase) genes, active after mannosidase I and before mannosidase II in the N-glycosylation pathway, did not affect worm viability under laboratory conditions (Zhu et al., 2004). It was suggested that environmental stress or knockout of other genes would be needed to influence viability (Zhu et al., 2004), in line with the finding that GnT I-dependent N-glycans are relevant for *C. elegans* interacting with pathogenic bacteria (Shi et al., 2006). Adding host immune components such as complement factors to the glyco-remodelled schistosomes could be a way to test the relevance of aberrant glycosylation in more challenging circumstances. Additionally, it would also be worth looking into the effects of mannosidase inhibition on parasite development by studying the effect of these inhibitors on developing larvae. After skin penetration, schistosomulas develop into adult worms over the course of six weeks. During this time, the (N-)glycan composition of the parasites changes considerably (Smit et al., 2015). Interfering with N-glycosylation during parasite maturation could have a larger impact on the developing organisms than in fully developed adult worms with a stable glycosylation pattern. Eventually, in view of the immunological effects triggered by schistosome glycans in diverse *in vitro* and *in vivo* models as well as the parasite’s complex development inside the host, a next important step would be to study glyco-remodelled schistosome infection of a host, for example by surgical transplantation of glyco-remodelled adult worms into a mouse (Wang et al., 2020).

Regarding the dynamics of N-glycosylation of *ex vivo* cultured adult worms over time, we observed that the N-glycan profile remained relatively stable during two weeks and was comparable to profiles reported before (Smit et al., 2015). However, N-glycans with two or three LN motifs (H5N5F1 and H6N5F1) decreased over time during culture. These N-glycans were found to be specific for freshly collected female parasites, as described before (Wuhrer et al., 2006). N-glycans with LN motifs are commonly found on egg glycoproteins (Khoo et al., 1997; Smit et al., 2015). During culture, the egg production by females quickly reduces (Michaels and Prata, 1968), which could explain the observed decrease in LN motifs. Additionally, we characterised the parasite’s tegumental N-glycan profile. As the tegument is the major site for parasite-host interactions, changes in glycosylation could have a major impact on these interactions (Acharya et al., 2021; Wilson and Jones, 2021). In freshly collected as well as PBS-control cultured schistosomes, up to90% of all N-glycans of the tegument were of the complex type, with a high proportion of LDN antenna, which is clearly different from the rest of the schistosome body N-glycosylation. Several complex N-glycan motifs are known ligands of C-type lectin receptors (CLRs), e.g. LDN as ligand for MGL, and mannose, LeX and LDN-F as ligand for MR or DC-SIGN (van Die et al., 2003; Meyer et al., 2005; van Liempt et al., 2006; Everts et al., 2012; van Kooyk et al., 2015; van der Zande et al.; Kuipers et al., 2023). Hypothetically, a shifted N-glycan profile could alter the interplay between glycan ligands and these CLRs present on immune cells such as dendritic cells and macrophages (van Vliet et al., 2008). While DC-SIGN, MGL and MR are all found on immature DC and macrophage subsets and on specific endothelial cells, the location of receptor-positive subsets often differs. A change in N-glycan motifs expressed by the parasite could therefore change local interactions with CLRs. Furthermore, dissection of the N-glycosylation of the tegument and stripped worm gives insight into the impact of the inhibitors on the different body parts of the schistosome. Although the uptake and diffusion of kifunensine and swainsonine by and through the schistosome body are unknown, the similarity in increase of mannose and hybrid N-glycans and decrease of LDN motifs between tegument and stripped worm indicate that the inhibitors penetrate effectively beyond the parasite’s surface.

LDN was the most common motif in the tegument of freshly collected adult parasites, with over 65% of all N-glycans containing this motif. Treatment with kifunensine and swainsonine led to a declining trend in the presence of LDN in the tegument, although not lower than 41.6% of the N-glycans, in conjunction with an increase in mannose glycans. It has been proposed that the normal turnover rate of the tegument is low (Saunders et al., 1987), which would explain the remaining high presence of LDN after mannosidase inhibition for 14 days. As it was suggested that rapid turnover is a mechanism to repair tegumental damage (Saunders et al., 1987), inducing tegumental damage may be an approach to test if N-glycosylation changes could progress towards a complete shift in tegument N-glycosylation. An additional question would be where the tegumental N-glycans originate from, since it has been reported that the tegument is void of endoplasmic reticulum (ER) and tegumental cell bodies contain little ER, while they are full of ribosomes (Wilson and Barnes, 1974). Thus, chemical α-mannosidase inhibition affects both the inner worm as well as its tegument, hypothetically altering glycan-CLR interactions, but slow turnover of the tegument could be an obstacle to achieve a complete shift in tegumental N-glycans in future studies.

Although N-glycosylation is a highly conserved process, differences in glycosyltransferases and glycosidases exist between species, preventing a direct comparison between mammalian cells and parasites. For example, sialic acids are amply present in mammalian N-glycans, but are absent in schistosomes (Cummings and Nyame, 1996; Smit et al., 2015). The availability of chemical inhibitors of (schistosome) N-glycosylation enzymes is limited. For instance, the fucosyltransferase inhibitor 2-fluoro-L-fucose, highly effective in mammalian cells, acts through the salvage pathway (Rillahan et al., 2012). This pathway converts fucose into GDP-fucose after which it can be incorporated into glycan molecules (Kizuka, 2022). Schistosome parasites do not possess the salvage pathway (Peterson et al., 2013) thereby excluding this fucosyltransferase inhibitor for further testing in schistosomes. Target-specific nucleic acid directed approaches, such as RNA interference or CRISPR (Hulme et al., 2022), may overcome the lack of chemical inhibitors additionally to α-mannosidase inhibitors, and avoid cross-reactivity of glycosylation-altering compounds to the host cells that would prevent host-parasite interaction studies.

To gain more insight into parasite-host interaction, *ex vivo* glyco-remodelling creates the possibility to use altered parasites for functional research, such as in immunological assays. Glycan remodelling of live parasites has already been applied for *Leishmania* proving the role of N-glycans in inducing protection in mouse immunisation studies (Gorczynski, 1986). For schistosomes, immunological research into parasite glycans has so far only been performed with isolated glycans or parasite products that were chemically or enzymatically altered. These methods are crucial for basic understanding of glycan involvement in parasite-host interaction, but fail to include the biological context and the dynamics of the parasite’s response to immune attack or other host induced effects. Glyco-remodelling of live *S. mansoni* overcomes this shortcoming, as whole parasites, excretory/secretory materials or extracts thereof can be used, preserving glycans in their native context. Future studies could endeavour to use glyco-remodelled schistosomes for co-cultures with host cells or *in vivo* experiments. Taken together, our study demonstrates the feasibility of developing a glyco-remodelled parasite, with the potential of employing in research into the impact of glycosylation on parasite development as well as parasite-host interaction studies.

## Supplementary Material

Supplementary Material

## Highlights

- Adult schistosome N-glycans change during culture with mannosidase inhibitors
- Kifunensine and swainsonine increase high mannose and hybrid glycans, respectively
- Tegument glycosylation profile is distinct from whole worm glycosylation profile
- No negative effects of the treatments on parasite motility or morphology
- Glycan remodelling of schistosomes for functional glycobiology studies is feasible
