# Supplementary material for: Glycan remodelling of live adult Schistosoma mansoni worms by chemical inhibition of α-mannosidases
Source: Int J Parasitol. Author manuscript; Available in PMC 2026 Sep 29. (PMC7619502; doi:10.1016/j.ijpara.2025.11.002)
Supplement: Supplementary file 1 [file EMS218271-supplement-Supplementary_Material.docx]

# Supplementary material

## Supplementary figure 1

**Supplementary figure 1 Overview of the experimental set-up (A) and N-glycan categories (B)**

d0: day 0, freshly collected worms; d4-14: culture day 4-14.

Supplementary figure 2

**Supplementary figure 2 Relative abundance of sex-specific N-glycans of adult schistosomes before and after 14 days of culture with and without α-mannosidase inhibitors**

The relative abundance of individual N-glycan structures was determined by dividing the signal-to-noise (S/N) ratio of the individual structure by the S/N ratio of all N-glycan structures combined per sample. The total N-glycan profile divided into glycan types is depicted for female and male worms that were freshly collected (d0), or cultured with PBS as control (d14 PBS), kifunensine (d14 kif) or swainsonine (d14 swain) (A). The structures H5N5F1 and H6N5F1 (B), containing LacNAc (LN; Galβ1–4GlcNAc), were mostly found in females, while H3N4F1 and H3N6F1 (C), containing the LacdiNAc (LDN; GalNAcβ1-4GlcNAc) motif, were predominantly found in male worms. H: hexose; N: hexosamine; F: fucose. Data of four biological replicates is included.

## Supplementary figure 3

**Supplementary figure 3 Relative abundance of N-glycan types before and after fourteen days of culture with and without α-mannosidase inhibitors**
The relative abundance of individual N-glycan structures was determined by dividing the signal-to-noise (S/N) ratio of the individual structure by the S/N ratio of all N-glycan structures combined per sample.

## Supplementary figure 4A

## Supplementary figure 4B

## Supplementary figure 4C

## Supplementary figure 4D

**Supplementary figure 4 MALDI-TOF MS analysis, HF-treatment and exoglycosidase digestions of N-glycans from adult schistosomes cultured for fourteen days with or without swainsonine.**

PNGase F-released N-glycans of PBS-control worms (A, C) and swainsonine-treated worms (B, D) were analysed by MALDI-TOF MS before and after hydrofluoric acid (HF) treatment and subsequent hexosaminidase digestion (A, B) , or before and after β1-4-galactosidase digestions (C, D). Arrows indicate the loss of fucose, N-acetylhexosamine or galactose as a result of HF-treatment or exoglycosidase digestion, respectively. Blue square: N-acetylglucosamine; green circle: mannose; yellow square: N-acetylgalactosamine; yellow circle: galactose; red triangle: fucose. M4-9: Man4-9GlcNAc2. For each condition a representative mass spectrum of two to four biological replicates is shown.

## Supplementary figure 5

**d14 swain**

**d14 kif**

**d14 PBS**

**d0**

**250 kD**

**150**

**100**

**75**

**50**

**37**

**25**

**20**

**Supplementary figure 5 Coomassie blue staining of worm (glyco)proteins**
An equivalent of two worms of each treatment was loaded on gel.

## Supplementary figure 6

**250 kD**

**150**

**100**

**75**

**50**

**37**

**25**

**20**

**d0**

**d14 PBS**

**d14 kif**

**d14 swain**

**d0**

**d14 PBS**

**d14 kif**

**d14 swain**

**Tegument**

**Stripped worm**

**Supplementary figure 6 Coomassie blue staining of tegument and stripped worm (glyco)proteins**
An equivalent of twenty worms (tegument) or two worms (stripped worm) of each treatment was loaded on gel.
